# Climate change, biodiversity loss, and Indigenous Peoples’ health and wellbeing: a systematic umbrella review protocol

**DOI:** 10.1186/s13643-023-02423-x

**Published:** 2024-01-02

**Authors:** Laura Jane Brubacher, Tara Tai-Wen Chen, Sheri Longboat, Warren Dodd, Laura Peach, Susan J. Elliott, Kaitlyn Patterson, Hannah Neufeld

**Affiliations:** 1 School of Public Health Sciences, University of Waterloo, 200 University Avenue West, Waterloo, ON N2L 3G1 Canada; 2 Department of Geography and Environmental Management, University of Waterloo, 200 University Avenue West, Waterloo, ON N2L 3G1 Canada; 3 School of Environmental Design and Rural Development, University of Guelph, 50 Stone Road East, Guelph, ON N1G 2W1 Canada; 4 Department of Geography and Environmental Studies, Wilfrid Laurier University, 75 University Avenue West, Waterloo, ON N2L 3C5 Canada

**Keywords:** Climate change, Biodiversity loss, Indigenous Peoples, Health, Wellbeing, Protocol, Umbrella review

## Abstract

**Background:**

Research that examines the intersections of Indigenous Peoples’ health and wellbeing with climate change and biodiversity loss is abundant in the global scholarship. A synthesis of this evidence base is crucial in order to map current pathways of impact, as well as to identify responses across the global literature that advance Indigenous health and wellbeing, all while centering Indigenous voices and perspectives. This protocol details our proposed methodology to systematically conduct an umbrella review (or review of reviews) of the synthesized literature on climate change, biodiversity loss, and the health and wellbeing of Indigenous Peoples globally.

**Methods:**

A multidisciplinary team of Indigenous and non-Indigenous scholars will conduct the review, guided by an engagement process with an Indigenous Experts group. A search hedge will be used to search PubMed®, Scopus®, Web of Science™, CINAHL (via EBSCOHost®), and Campbell Collaboration databases and adapted for use in grey literature sources. Two independent reviewers will conduct level one (title/abstract) and level two (full-text) eligibility screening using inclusion/exclusion criteria. Data will be extracted from included records and analyzed using quantitative (e.g., basic descriptive statistics) and qualitative methods (e.g., thematic analysis, using a constant comparative method).

**Discussion:**

This protocol outlines our approach to systematically and transparently review synthesized literature that examines the intersections of climate change, biodiversity loss, and Indigenous Peoples’ health and wellbeing globally.

**Systematic review registration:**

This protocol was registered with the International Prospective Register of Systematic Reviews (PROSPERO) on April 24, 2023 (registration number: CRD42023417060).

**Supplementary Information:**

The online version contains supplementary material available at 10.1186/s13643-023-02423-x.

## Background

For Indigenous Peoples^1^ worldwide, the health of the land and the community are synonymous [1, 2]. Amid significant cultural and linguistic diversity, as well as the diverse economic, social, and political contexts in which Indigenous Peoples live, a common denominator is shared ongoing and historical traumas related to settler/industrial colonialism [3, 4]. Colonial dispossession of Indigenous lands, and forced assimilation associated with urbanization and industrial resource extraction, has reduced Indigenous Peoples’ access to physical environments, and also the relationships or social environments required to sustain them [2, 5, 6]. These processes have also invariably contributed to the degradation of the land and the decline in the overall health of environments and species therein [7]. Colonialism is a fundamental driver, then, of environmental degradation, loss of species, and climate change, but also Indigenous health inequities [8–11].

Indeed, Indigenous Peoples are among those who have contributed least to the problems of climate change, environmental degradation, and biodiversity loss, yet are amongst those experiencing the greatest impacts [12]. With livelihoods, knowledge systems, and ways of being intrinsically tied to land and place, Indigenous Peoples are disproportionately affected by the climate crisis [13–15]. Research demonstrates that changes to the land affect all facets of Indigenous Peoples’ health and wellbeing, whether physical, emotional, mental, or spiritual [16–18]; however, the gendered health aspects of climate change among Indigenous Peoples have been underexplored, particularly related to gender-diverse identities. Overall, impacts on Indigenous Peoples’ health and wellbeing have been characterized as having three transversal “levels” or dimensions: primary (direct physical health impacts), secondary (related to ecosystem changes), and tertiary (related to culture-wide changes) [19], as well as gendered dimensions [20, 21].

Within this context, Indigenous Peoples play a fundamental role in protecting biological diversity and preventing environmental degradation globally [22]. In response to climate change, and ecological crises more generally, Indigenous Peoples, Nations, and organizations are developing community-led monitoring and adaptation strategies that draw on Indigenous knowledge and science to advance the health and wellbeing of communities, lands, waters, and non-human species [23–27]. Indigenous Peoples are also crucial actors in global climate-policy processes and the development of frameworks for climate action and biodiversity conservation, giving voice and direction through, for instance, the Intergovernmental Panel on Climate Change (IPCC), Intergovernmental Science-Policy Platform on Biodiversity and Ecosystem Services (IPBES), and global mechanisms such as the United Nations Permanent Forum on Indigenous Issues (UNPFII), and Expert Mechanism on the Rights of Indigenous Peoples (EMRIP). Recent reports from these bodies increasingly call for equity considerations and Indigenous rights, knowledge, and perspectives to drive future action [19, 28–30]. In light of these, and ongoing community-level and global processes, there is a need to understand alignment between the work that has been done and calls for future action, across literatures, scales, and geographies.

Research at the interface of Indigenous health and wellbeing, climate change, and biodiversity loss is prolific; yet, a synthesis of this evidence base is crucial, in order to map current pathways of impact, highlight gaps requiring further investigation, and identify responses across the global literature that advance Indigenous health and wellbeing, while centering Indigenous voices and perspectives. Given the abundance of both primary and secondary research at this interface, an umbrella review (or review of reviews) is the chosen and appropriate methodology for generating analytic insights across already-synthesized evidence [31, 32].

### Research question and objectives

This systematic umbrella review will be guided by the overarching question: What are the pathways through which climate change and biodiversity loss intersect with Indigenous health and wellbeing,^2^ as reported in the global secondary literature? Based on the synthesized literature retrieved and analyzed, we aim to address a number of interrelated objectives including:

1. To characterize the extent, range, and nature of secondary literature on climate change, biodiversity loss, and Indigenous health and wellbeing globally;
2. To examine the connections between climate change, biodiversity loss, and Indigenous health and wellbeing, characterizing the proximal, intermediate, and distal impacts^3^ within;
3. To explore the gendered impacts of climate change and biodiversity loss on Indigenous health and wellbeing; and
4. To identify responses to climate change and biodiversity loss that also advance Indigenous health and wellbeing.

## Methods

### Research design and guiding frameworks

This protocol was registered in the International Prospective Register of Systematic Reviews (Registration #: CRD42023417060) and developed in accordance with the Preferred Reporting Items for Systematic Reviews and Meta-Analyses (PRISMA-P) guidelines, as well as methodological recommendations for umbrella reviews [31]. The completed PRISMA-P checklist is available in Additional file 1.

This umbrella review will be conducted by a multidisciplinary team of Indigenous (*n* = 2) and non-Indigenous scholars (*n* = 6) from the University of Waterloo (UW) and University of Guelph, Canada, with support from the Waterloo Climate Institute, a UW research librarian, and a WHO Technical Officer. Our team has collective expertise in Indigenous health and wellbeing, climate change and climate-health literacy, global environmental and public health, health geography, food environments and nutrition, Indigenous planning, environmental governance and policy, resource management, and knowledge syntheses. A group of Indigenous Experts, convened by the WHO and with representation across the WHO’s member regions, has been engaged in the development of the research questions and design, and will continue to guide the data extraction and analysis procedures, as well as share perspectives on the emerging findings.

As a team of Indigenous and non-Indigenous researchers from within Canada, we approach this work with a vested interest in finding pathways that strengthen Indigenous wellbeing and mirror community values. We aim to take a strength-based approach—that is, to highlight the social and ecological dimensions or determinants of environments and to illustrate innovative approaches towards health and wellbeing, community-level protective factors, and Indigenous-led adaptation strategies to climate and biodiversity threats. Additionally, we recognize the tendency of knowledge synthesis methodologies to centre bibliometric, quantitative approaches over other knowing practices and positivism over other worldviews [35]. As such, this review will prioritize critical, reflexive, and collective engagement with the literature as another means of knowing the evidence [35].

### Search strategy

The search strategy for databases and grey literature has been designed collaboratively by members of the research team, with the assistance of a UW librarian, and refined through an engagement process with a group of Indigenous Experts with global representation.

#### Database search

The following databases will be searched to capture a range of published literature across disciplines: PubMed®, Scopus®, Web of Science™, and CINAHL (via EBSCOHost®). No restrictions on the geographic location, date of publication, or language will be applied, with the exception of limiting our search to English records in Scopus® to retrieve a manageable volume of records. The type of record will be limited to academic journals in Scopus® (for volume) and CINAHL® (which indexes a higher proportion of non-academic sources, e.g., magazine articles, teaching curricula). The search hedge contains terms related to Indigenous Peoples, health, climate change, and reviews of reviews (Table 1). Terms were retrieved through a snowball search of the reference lists of relevant articles on the climate-Indigenous health nexus and identification of terms used for the concepts of “Indigenous Peoples”, “health”, and “climate change”; search strategies of review articles on this topic (e.g., Harper et al., [36]) and for reviews of reviews (e.g., Kinchin et al., [37]); and through consultation with a UW research librarian. Further, a detailed search hedge on the topic of climate change, developed by librarians for use by the Waterloo Climate Institute, as well as relevant research guides produced by the University of Alberta library [38] and McMaster University Health Information Research Unit [39] were consulted to further adapt and refine the search hedge.

**Table 1:** Search hedge developed for the Web of Science™ multidisciplinary database and subsequently adapted

Concept/component	Search terms
Indigenous Peoples	Aasax OR Aboriginal* OR “Aboriginal-Malay” OR Aborigine OR […]^a^ **AND**
Health	health OR “one health” OR wellness OR wellbeing OR well-being OR disease* OR morbidity OR mortality OR illness* OR infect* OR death OR injur* OR medical OR disorder **AND**
Climate change	“climate change*” OR “climatic change*” OR “environmental change*” OR "environmental loss*" OR "environmental degradation" OR "environmental dispossession" OR “changing climate*” OR “ecosystem change*” OR “ecological change*” OR “climate risk*” OR “climatic risk*” OR “extreme climate*” OR “climate uncertaint*” OR “climate variability” OR “climatic variability” OR “climate disaster” OR “climate resilience” OR “carbon footprint” OR “global warming” OR “earth warming” OR “global temperature” OR “greenhouse effect” OR “greenhouse gas*” OR GHGE OR “carbon emission*” OR carbon OR decarbonization OR holocene OR anthropocene OR cryospher* OR atmospher* OR biodiversity OR "biodiversity loss" **AND**
Review of reviews	review* OR “metaanalysis” OR metaanalysis OR "knowledge synthesis" OR "evidence synthesis" OR overview

^a^“Indigenous Peoples” search terms (including umbrella terms “Aboriginal*” and “Indigenous” as well as specific people groups/nations) are adapted from [40] and abbreviated here. The full-search hedge can be found in Additional file 2.1

In addition to the above databases, the Campbell Collaboration database will be searched using umbrella terms for “Indigenous Peoples” and “Climate Change” to identify health-related reviews that may be relevant to include. Complete search hedges, adapted to each individual database, can be found in Additional file 2.1. Additionally, the following journals will be hand-searched for potentially relevant articles (based on title; published 2013-present) to explore the sensitivity of the search hedge and to retrieve any additional articles: *The Journal of Climate Change and Health, Environmental Health Perspectives, The Lancet Planetary Health, International Journal of Circumpolar Health, Anthrosource, AlterNative,* and the *International Journal of Indigenous Health* (further detail in Additional file 2.2).

An initial test search was conducted on February 9, 2023. This search will be updated to retrieve literature published within the full calendar year prior to publication.

#### Grey literature search

A search will be conducted for available information within the public domain (grey literature), guided by an adapted framework developed by UW colleagues [41]. In particular, this search will draw on relevant Indigenous health-related information from the 2022 Intergovernmental Panel on Climate Change (IPCC) report [19], the Global Assessment Report on Biodiversity and Ecosystem Services [28], The Health Argument for Climate Action [29], and the WHO’s State of Knowledge Review on Biodiversity and Health [30]. Country reports of the Special Rapporteur on the Rights of Indigenous Peoples may provide more detailed country-specific information to address potential gaps in geographic coverage from the database searches [42].

Overall, the grey literature search will involve three main strategies, including, but not limited to the following:

- *United Nations (UN) Database Search*: search websites of UN agencies and organizations, e.g., UN digital library, WHO IRIS, UNDESA Special Rapporteur Reports, IPBES, ILO, FAO, WFP, and IPCC;
- *Targeted Search by Geographic Region:* search databases that may be subject-specific (e.g., Indigenous health, climate change) or collections from specific regions less represented in the published literature, including sub-Saharan Africa, Middle East, Southeast Asia, Oceania, Australia, New Zealand, Europe, Latin America, and Caribbean (e.g., Africa Portal, Indigenous Studies Portal); and
- *Contact Knowledge Experts:* identify Indigenous Experts who may be able to recommend published or unpublished records, with attention towards regions less represented in the published literature.

Citations for all retrieved records will be exported in .ris format from the databases into *Covidence*™ for automatic de-duplication and subsequent eligibility screening. Retrieved grey literature will be uploaded in full-text format to *Covidence*™ and screened separately.

### Article selection

#### Inclusion and exclusion criteria

**Fig. 1:**
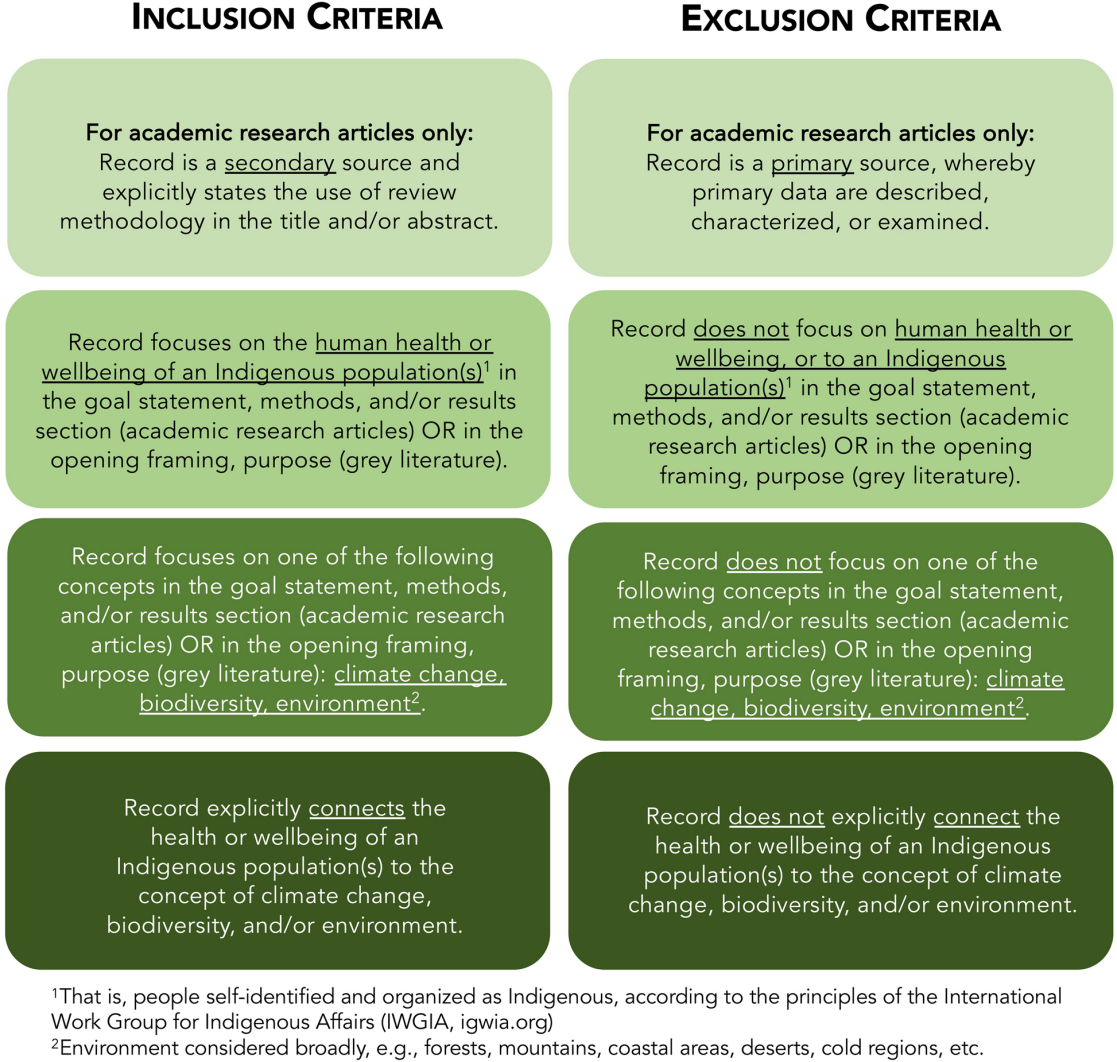
Inclusion and exclusion criteria for academic research articles and grey literature

To be included, retrieved records from the database and grey literature searches must explore, evaluate, or examine a relationship(s) between Indigenous health (as defined in the subsequent paragraph) and climate change, biodiversity, or environment (Fig. 1). That is, records that discuss Indigenous Peoples’ health or wellbeing or climate change or biodiversity or the environment will be excluded, unless explicit connections are made between these concepts. Records that focus on resource extraction (rather than climate change) as the antecedent to health/wellbeing impacts will be excluded, as will records that synthesize climate-health literature with a particular focus on research methodologies or conceptual approaches in this scholarship (Additional file 2.3). Records from database searches must also be secondary sources.

Indigenous Peoples’ conceptions of health and wellbeing are multidimensional. Wellbeing is a complex and hard-to-measure concept [43]. Lived experience across the lifespan, and the potential for it to affect individuals and communities, needs to be considered. For the purposes of this review, wellbeing will be situated as individual and inter-generational; embedded in the land [33]; all our relations; across the continuum of past, present, and future; and across dimensions of the body (physical), heart (emotional), mind (intellectual), and spirit (spiritual) [34]. Elements of autonomy (self-determination), connection (family, place), along with culture (language, identity, knowledge) [44, 45] also factor in as they are central to the experiences, priorities, and needs of Indigenous Peoples and specific to local contexts.

#### Level 1 (title/abstract) screening

Two reviewers will independently screen the title/abstract of each database record according to the pre-established eligibility criteria. Specifically, a stacked screening form will be used, whereby if an inclusion criterion is not met, subsequent criteria will not be assessed. Records meeting all inclusion criteria (e.g., both reviewers assign an assessment of “yes” or “unclear” to all criteria) will proceed to level two (full-text) screening. Records not meeting all inclusion criteria will not proceed to level two screening. Conflicts between reviewers as to the inclusion/exclusion of a given record will be discussed and resolved by consensus in collaboration with a third reviewer. A Cohen’s Kappa coefficient (κ) will be calculated to indicate the level of agreement between reviewers.

#### Level 2 (full-text) screening

The full text of records meeting all inclusion criteria will be retrieved and uploaded into *Covidence*™ for level two (full-text) screening. Two independent reviewers will apply the same stacked screening form to each full text (“unclear” will not be an option in level two). Additionally, in level two, whether a record focuses on human health or wellbeing of an Indigenous population(s), or on the concepts of climate change, biodiversity, or environment, will be defined as the presence of at least one paragraph of results or discussion. Conflicts between reviewers as to inclusion/exclusion will be discussed and again resolved by consensus, with the support of a third reviewer. Records meeting all inclusion criteria based on full-text screening will proceed to data extraction and analysis.

#### Weighted criteria

In an effort to increase the visibility of and center literature from geographic regions or specific populations less represented in the peer-reviewed literature, we will apply weighted criteria when resolving conflicts between reviewers. That is, provided that all other criteria are met, if an article is not specifically focused on Indigenous Peoples but focuses on regions/populations less represented, we will lean to the side of inclusion. This weighted criteria will apply to the following regions: sub-Saharan Africa, Middle East, Southeast Asia, Oceania, Latin America, and the Caribbean [46].

### Data extraction and analysis

The proposed data extraction form (Table 2) will be piloted by two independent reviewers who will each randomly select an included article from which to extract data pertaining to the proposed domains. If any domains are unclear, or further categories are required within domains, the extraction form will be revised accordingly. Data will then be extracted from all included articles into this piloted extraction form using *Covidence*™. This process will again be conducted by two independent reviewers to reduce selection bias and ensure interrater reliability [47]. Extracted data will include basic information about the record (e.g., author name(s), name of record, year of publication, geographic location of study), type of record (study methodology for review article; category of grey literature^4^), and information about the stated aim/purpose of the record and characteristics/demographic of the population(s) being discussed.

**Table 2:** Proposed data extraction template, indicating domains for which reviewers will extract data

Research objective	Data extraction domains
To characterize the extent, range, and nature of secondary literature on climate change, biodiversity loss, and Indigenous health and wellbeing globally	• Name of record • Year of publication • Geographic location(s) covered, if applicable • Indigenous Peoples (Nations, groups, organizations) • Review methodology (systematic, scoping, critical review, meta-analysis, other) • Number of databases searched • Date range of database searches • Date range of included primary studies • Types of primary studies included in the review (quantitative, qualitative, mixed-methods, other) • Number of primary research records reviewed • Discipline • Theory/framework/model engaged (e.g., EcoHealth, One Health, nature-based solutions) • Purpose/aim of the record
To examine the connections between climate change, biodiversity loss, and Indigenous health and wellbeing, characterizing the proximal, intermediate, and distal impacts within	• Relationships examined between concepts (e.g., climate change AND Indigenous health broadly; biodiversity loss AND Indigenous mental health) and rationale for this examination • Proximal impact(s) (if applicable) • Intermediate impact(s) (if applicable) • Distal impact(s) (if applicable) • Specific impacts on biodiversity • Scale of impact (e.g., individual, household, community, population, regional, national, global) and explanation • Key findings/conclusions about the relationships studied • Any further comments/observations/relevant data
To explore the gendered impacts of climate change and biodiversity loss on Indigenous health and wellbeing	• Summary of findings regarding gendered impacts • How sex/gender are discussed in this context
To identify responses to climate change and biodiversity loss that also advance Indigenous health and wellbeing	• Recommended strategies to address impacts; when applicable, categorized as follows: • Community-level or population-level • Regional-level or global-level • Policy responses • Future research

Building from and adapting the three-level framework [19], data will also be extracted according to proximal, intermediate, and/or distal impacts of climate change and biodiversity loss, as identified by authors: (1) proximal health effects of climate change and biodiversity loss, including the immediate physical effects on human health and wellbeing; (2) intermediate effects related to ecosystem changes; and (3) distal effects related to culture-wide changes (e.g., malnutrition due to climate-driven changes in food systems, mental health challenges related to cultural losses). Data pertaining to the gendered impacts of climate change and biodiversity loss will also be extracted. See Additional file 2.4 for a modified data extraction tool for grey literature records.

Data extracted from the included articles will be categorically synthesized and tabulated. Basic descriptive statistics (e.g., proportions, means) will be calculated to quantify the extent and range of secondary literature. The nature of this literature will be characterized qualitatively, whereby extracted data will be analyzed thematically, using a constant comparative method, to identify cross-cutting themes [48]. Preliminary interpretations of the data will be reviewed by the Indigenous Experts group, to enrich the analyses, and enhance rigor and validity. This process may also involve collectively identifying gaps (e.g., in geography; themes; representation) in the synthesized literature, some of which may be addressed through an iterative, targeted search and integration of the grey literature.

### Quality appraisal

We will conduct a quality appraisal of published review articles included in the umbrella review, using an adapted version of the *Critical Appraisal Skills Programme (CASP)* tool for Systematic Reviews and *JBI Checklist for Systematic Reviews and Research Syntheses* for other review methodologies. Grey literature will be assessed using the *AACODS tool*. In addition to questions related to general methodological quality, we will assess the following, drawing from the assessment of external validity in reviewed articles conducted by Jones et al. [22]:

- Degree of attentiveness to/recognition of colonialism as an antecedent to and driver of the climate-health pathways being explored, as well as its historic and ongoing impact on Indigenous Peoples’ health and wellbeing
- Level of Indigenous Peoples’ involvement in the research (for published literature) or initiative/policy (for grey records) (e.g., co-design, identification of research question(s), contribution of Indigenous knowledge, perspectives, or values to the interpretation of findings)
- Extent to which a record discusses the relevance of the findings to Indigenous Peoples’ priorities and processes
- Extent to which a record centers Indigenous-led strategies and responses, and focuses on advancing Indigenous health and wellbeing

Each of these quality appraisal domains adapted from Jones et al. [22] will be scored as high, medium, low, or unsure. A minimum of two independent reviewers will appraise all published records and discuss when conflicts arise. We will not exclude articles based on low scores, although quality appraisal scores will be reported in a supplementary file to the review. Articles that reported engagement with Indigenous Peoples in the interpretative process will be particularly emphasized in the results.

## Discussion

This umbrella review will examine the global scholarship on climate change, biodiversity loss, and Indigenous Peoples’ health and wellbeing, with a particular focus on the pathways through which impacts are experienced; the gendered nature of these impacts; and Indigenous-led adaptation strategies, research, and action that advances health and wellbeing. This work is timely, given the burgeoning primary literature at the climate-health nexus and the critical need for synthesized evidence to inform global climate priorities and action.

### Strengths and limitations

A key strength of this review is the collective experience and topical expertise of our research team, as well as the focused engagement process with an Indigenous Experts group to guide, inform, and shape the work. Being an umbrella review, this study will be limited to data that has already been synthesized. As such, we may lessen the depth and nuance of inquiry that could come from an examination of primary literature at the climate-health nexus, and inclusion of community-level insights and perspectives. In an effort to address these limitations, we will aim to integrate grey literature that highlights community voices and fills other gaps in inquiry.

Relatedly, the design of this review relies on written knowledge of the impacts of climate change and biodiversity loss to Indigenous health and wellbeing—much of which is generated within Western research frameworks and epistemologies—and precludes the inclusion of other forms of knowledge, knowledge-sharing, and knowledge co-creation. Centering lived experiences of climate change and biodiversity loss impacts, and privileging Indigenous knowledge and Indigenous science, can further understanding of these impacts. This is a critical area of future research and action, because:

“We’re fighting for soil, land, food, trees, water, birds. We’re fighting for life.”—Gregorio Mirabal, Indigenous leader and coordinator of the Confederation of Indigenous Organizations of the Amazon Basin (COICA).

### Supplementary Information

**Additional file 1.** PRISMA-P Checklist.

**Additional file 2: Additional file 2.1.** Complete search strings used for PubMed, CINAHL®, Web of Science™, and Scopus®. **Additional file 2.2.** Approach for hand searching of journals, to explore search hedge sensitivity for the database search. **Additional file 2.3.** Examples of topics included/excluded. **Additional file 2.4.** Data extraction tool for grey literature sources.

## Data Availability

Not applicable.

## Abbreviations

EMRIP: Expert Mechanism on the Rights of Indigenous Peoples
IPBES: Intergovernmental Science-Policy Platform on Biodiversity and Ecosystem Services
IPCC: Intergovernmental Panel on Climate Change
IWGIA: International Work Group for Indigenous Affairs
PRISMA-P: Preferred Reporting Items for Systematic Reviews and Meta-Analyses, Protocol Extension
UNPFII: United Nations Permanent Forum on Indigenous Issues
UW: University of Waterloo
WHO: World Health Organization

## Footnotes

1 We refer to Indigenous Peoples as people self-identified and organized as Indigenous, according to the principles of the International Work Group for Indigenous Affairs (iwgia.org), an International NGO with observer status at the United Nations.

2 For the purposes of this review, our definition and operationalization of Indigenous health aligns with Indigenous conceptualizations of health as inclusive of mental, emotional, spiritual, and physical wellbeing and intrinsically tied to the land, land-based livelihoods, and relationships [33, 34].

3 Drawing from and adapting the three-level framework of climate-change impacts on health proposed by Neufeld et al. [19], whereby proximal impacts refer to direct impacts to physical health; intermediate impacts are those related to broader ecosystem changes; and distal impacts relate to culture-wide changes. These “levels” are considered transversally or cyclically, not hierarchically. We have reframed primary, secondary, and tertiary as proximal, intermediate, and distal, respectively.

4 For example: working paper, report, or policy brief.
