# Supplementary material for: Climate change, biodiversity loss, and Indigenous Peoples’ health and wellbeing: a systematic umbrella review protocol
Source: Syst Rev. 2024 Jan 2;13:8. doi: 10.1186/s13643-023-02423-x (PMC10759611; doi:10.1186/s13643-023-02423-x)
Supplement: Supplementary file 2 — Additional file 2: Additional file 2.1. Complete search strings used for PubMed, CINAHL®, Web of Science™, and Scopus®. Additional file 2.2. Approach for hand searching of journals, to explore search hedge sensitivity for the database search. Additional file 2.3. Examples of topics included/excluded. Additional file 2.4. Data extraction tool for grey literature sources. [file 13643_2023_2423_MOESM2_ESM.docx]

**Additional file 2**

**Additional File 2.1 –** Complete search strings used for PubMed, CINAHL®, Web of Science™, and Scopus®

**Web of Science:**

| **Concept** | **Search Terms** |
| --- | --- |
| **INDIGENOUS PEOPLES** | (Aasax OR Aboriginal* OR “Aboriginal-Malay” OR Aborigine OR Achi OR Achuar OR Adibashi OR Adivasi OR Adivasis OR Afar OR Ainu OR Aka OR Akawai OR Akha OR Akie OR Akoula OR Akurio OR Akwoa OR “al-Kaabneh” OR “al-Asarmeh” OR “al-Ramadin” OR “al-Rshaida” OR “Alaska Native*” OR Aleut OR Alutor OR Amazigh OR Ambo OR “American Indian*” OR Ameridian* OR Amuesha OR Anak OR “Andean Kichwe” OR Andoa OR Andorrans OR Angaite OR Anikhwe OR Anu OR Arara OR Arawak OR “Arawak-Taino” OR Arwak OR Ashaninka OR Atayal OR Austronesian OR “Ava Guarani” OR Awajun OR Awa OR Awakateco OR Aweer OR Ayeoreo OR Aymara OR Ayoreo OR Aztec OR Baaka OR Baantonu OR Babi OR Bahnar OR Babongo OR Bacwa OR Bagame OR Bagombe OR Bagyeli OR BaGyeli OR Bajuni OR Baka OR Bakgalagadi OR Bakola OR Bakongo OR Bakoya OR Balala OR Bambara OR Bambuti OR Bantu OR Barabaig OR Bariba OR Barimba OR Basarwa OR Bassari OR Batwa OR Bawarwa OR BaWka OR Bawn OR BaYeyi OR Bedzam OR Benet OR Berabis OR Berawan OR Berber OR Berbers OR Bidayuh OR Bigombe OR Biharis OR Bilma OR Bisayah OR Bobo OR Boeschs OR Bofi OR Boni OR Bonis OR Boranna OR Boro OR Bororo OR Boruka OR Botsarwa OR Bozo OR Brao OR Bribri OR “Bri Bri” OR Brunca OR Bugakhwe OR Bulu OR Bumipeuteras OR Bunak OR Bunun OR Bwiti OR Cabecar OR Cacaopera OR Campeche OR Carib OR Caribs OR “Ch’orti” OR Chachi OR Chaima OR Chakma OR Chalchiteco OR Chamorro OR Chamorru OR Chamorous OR “Chao-Khao” OR “Chao Ley” OR Charrua OR Chelkancy OR Chiapas OR Chibcha OR Chibchense OR Chipaya OR Chiriguano OR Chiquito OR Chiquitano OR Chorotega OR Chorti OR Cofan OR Chuaa OR Chuj OR Chukchi OR Chulymcy OR Chuvancy OR Ciboney OR “Ciboney-Taino-Arawak” OR “Cocama-Cocomilla” OR Colla OR Copts OR Cotier OR Cree OR Cumanagoto OR Dabou OR Dagiri OR Dahalo OR Danisi OR Daroobe OR Datoga OR Daza OR Degar OR Deti OR Diaguita OR Dinka OR Dioula OR Ditammari OR Dogon OR Dolgan OR Doma OR Dukha OR Dusun OR Ebrie OR “egga hodaabe” OR Elmolo OR “El Mono” OR Embera OR Emerillon OR Ency OR Endorois OR “Enlhet Norte” OR Enxet OR “Enxet Sur” OR Epera OR Eskimo OR “Ese Eja” OR Evenk OR Ewondo OR Fatukuku OR “First Nation*” OR “Forest dwell*” OR Fuegian OR Fulani OR Fulbe OR Galibia OR Galibi OR Garifuna OR Gaoshan OR Gio OR Guadalcanese OR Guana OR Guaicuru OR Guarani OR “Guarani Mbya” OR Guyami OR Guaymi OR Guerrero OR Gurani OR Guransi OR Gurung OR “G//ana” OR “G/wi” OR “Gwich’in” OR Hadzabe OR Hadza OR Haida OR Herero OR Hidalgo OR “Hill People” OR “Hill Person” OR Hmong OR Hoa OR Huambisa OR Huastec OR Hui OR Huetar OR Hutu OR Iban OR Igotot OR “Ik” OR Imazighn OR Imazighen OR Indigenous OR Ingarico OR Inuit OR Inupiat OR Inuvialut OR Iroquoian OR Itelmen OR “Itza’” OR Ixil OR Jacalteco OR “Jahalin Bedouin” OR Jarai OR Jivi OR Jumma OR “Ju’hoansi” OR “K’iche” OR “Ka Pei Aina” OR Kachin OR Kaiowa OR Kalanga OR Kalina OR “Kalina-go” OR Kalinago OR “Kalinago-Taino” OR “Kali’na” OR Kamchadal OR Kanak OR “Kanaka Maoli” OR Kanuri OR Kaqchikel OR Karamajong OR Karenni OR Kavalan OR Kayapo OR Kawashkar OR Kayan OR Kazakh OR Kedayan OR Kelait OR Kenyah OR Kereki OR Kety OR “Khali’nago” OR Khamu OR Khanty OR Khengs OR “Khmer Krom” OR Khoekhoe OR “Khoe-San” OR Khoikhoi OR Khoisan OR Khomani OR “Khudro Nrigoshthhi” OR Khumi OR Khwe OR Khyang OR Kichwas OR Kipsigis OR Kirdi OR Koba OR Koryak OR Krio OR Krohn OR Kua OR Kumandincy OR Kuna OR Kuy OR Kwisi OR Lahu OR Lao OR “Laotian Tribes” OR Lenca OR Lickanantay OR Limbu OR Lisu OR Livs OR Lobi OR Lokono OR Loma OR Lua OR Lumad OR “Lunda-Chokwe” OR Lushai OR Maasai OR Macourai OR Macuxi OR Macuzi OR Magar OR Makasae OR Makuxi OR Malagasy OR Malakote OR Malay OR “Malayo-Polynesian” OR Maleku OR Mangyan OR Mani OR Mano OR Mansi OR Maori OR Mam OR Manjo OR Marma OR “Marsh Dwellers” OR Mapuche OR Maskoy OR “masyarakat adat” OR Mataco OR “Mataco Matguayo” OR Matagulpa OR Maya OR “Maya Chorti” OR Mayagna OR Mbanderu OR Mbini OR Mbororo OR Mbukushu OR Mbundu OR Mbuti OR Mbri OR Mbya OR Mdendjele OR Melanesian OR “Melanesian-Papauan” OR Mestico OR Mestizo OR Merina OR Metis OR Miao OR Mien OR Mikaya OR Miskito OR Miskitu OR Misquito OR Mixte OR Mnong OR Mogeno OR “Mon-Khmer” OR Montagnards OR Mopan OR Moxeno OR Mozabite OR Mpukushu OR Mru OR Muong OR Murut OR“N/oakhwe” OR “N’guigmi” OR Nagas OR Nahoa OR Nahua OR Nahuatl OR Nama OR Nambiquara OR Nanaicy OR Nandeva OR “Nandevi Guarani” OR Naro OR “Naso Tjerdi” OR Native* OR “Native American*” OR “Native Hawai’ian*” OR Negidalcy OR Negeri OR Negrito OR Nemadi OR Nenets OR Nganasan OR Ngabe OR Ngobe OR “Ngobe-Bugle” OR Nivkhy OR Nuer OR “Nyaneka-Nkumbi” OR Oaxaca OR Ocanxiu OR Ogiek OR Ogoni OR Ojibway OR Okinawans OR “Orang Asli” OR Orochi OR Oroki OR Otomi OR Ovimibundu OR Oyampi OR “Pai Tavytera” OR Paiwan OR Palenqueros OR Palikur OR Pankho OR Patamona OR Pech OR Pemon OR Peul OR Peulh OR Penan OR Piaroa OR “Ping Pu” OR Pipil OR Pocomam OR Pokot OR Poqomam OR “Poqomchi’” OR Puebla OR Punan OR Puyuma OR “Q’anjob’al” OR “Q’eqchi” OR Qawasqar OR Qicaque OR Quechua OR Quenchua OR “Quintano Roo” OR Qom OR Rai OR Raisales OR Rakhine OR Rama OR Rapanui OR “Rapa Nui” OR Raute OR Rhade OR Roraima OR Rotumans OR Rukai OR Saami OR Sabaot OR Saharawis OR Saisyat OR Sakapulteco OR Sakizaya OR Sami OR (San AND Africa) OR Sanapan OR Sanapana OR Sandawe OR “Santa Rosa Carib” OR Sanya OR Saramancas OR (“Scheduled Tribes” AND India) OR Secoya OR Sediq OR Selkup OR Semang OR Sengwer OR Senoi OR Shan OR Sherpa OR Shipibo OR “Shipibo-Conibo” OR Shiwiar OR Shorcy OR Shua OR Shuar OR Siona OR Sipakapense OR Soioty OR “South Sea Islander*” OR Stieng OR “Sumu-Mayangna” OR Sutiaba OR Tachangya OR “Tai-Kadai” OR Taino OR “Taino-Kalingo” OR Tamang OR Tampuan OR Tapeba OR Tapebo OR Tareno OR Taurepang OR Tawahka OR Tazy OR Teda OR Teenek OR Teko OR Tektiteko OR Telengity OR Teleuty OR Temenbe OR Teribe OR Tesker OR Thakali OR Tharu OR Thao OR Tikuna OR Tikigaq OR Tirio OR Toba OR “Toba Maskoy” OR Tofolar OR Tolai OR Toloupan OR Tomarao OR Topnaars OR “Torres Straight Islander*” OR Totonac OR Toubou OR Truku OR Tsexakhwe OR Tripura OR Tsaatan OR Tsachila OR Tsou OR Tsumkwe OR Tshwa OR Tuareg OR Tuaregare OR Tubolar OR Tubu OR Tugen OR Tukano OR Tupi OR Tutong OR Tutsi OR “Tuvin-Todjin” OR Twa OR Tyua OR “Tz’utujil” OR Tzeltal OR Tzotzil OR Uchay OR Udege OR Ulchi OR “Ureueu-Wau-Wau” OR Uru OR Uspanteko OR Vadda OR Vadema OR Vai OR Veddhas OR Veps OR Vyadha OR “Waaniy-a-Laato” OR Waata OR Wadoma OR Wagashi OR Wapaichana OR Waorani OR Wapixana OR Warao OR Warrau OR “Warrau Wayana” OR Wayampi OR Wayana OR Wayeyi OR Wayuu OR Wichi OR Wodaabe OR Wounaan OR Xinka OR Yaaku OR Yami OR Yamana OR Yanomami OR Yukpa OR Yvytoso OR Zamuco OR Zapara OR Zapotec OR “!Xoo” OR “//’Xauesi” OR “/Xaisa” OR “‘Akateco” OR ([Indigenous OR Aboriginal OR Native] AND [Ache OR Algonquin OR Amis OR Bedouin OR Bugle OR Bushmen OR Dakota OR Dan OR Fang OR Herder OR Herdsmen OR Indian* OR Karen OR Maroon OR Mohawk OR Mon OR pastoralist* OR Palestinian OR Papua OR Pear OR Potters OR Pygmy OR Pygymy OR Rade OR Roma OR Sab OR Squamish OR Tay OR Trio OR Yucatan]))  **AND** |
| **HEALTH** | (health OR “one health” OR wellness OR wellbeing OR well-being OR disease* OR morbidity OR mortality OR illness* OR infect* OR death OR injur* OR medical OR disorder)  **AND** |
| **CLIMATE CHANGE** | (“climate change*” OR “climatic change*” OR “environmental change*” OR "environmental loss*" OR "environmental degradation" OR "environmental dispossession" OR “changing climate*” OR “ecosystem change*” OR “ecological change*” OR “climate risk*” OR “climatic risk*” OR “extreme climate*” OR “climate uncertaint*” OR “climate variability” OR “climatic variability” OR “climate disaster” OR “climate resilience” OR “carbon footprint” OR “global warming” OR “earth warming” OR “global temperature” OR “greenhouse effect”  OR “greenhouse gas*” OR GHGE OR “carbon emission*” OR carbon OR decarbonization OR holocene OR anthropocene OR cryospher* OR atmospher* OR biodiversity OR "biodiversity loss")  **AND** |
| **REVIEW OF REVIEWS** | (review* OR “metaanalysis” OR metaanalysis OR "knowledge synthesis" OR "evidence synthesis" OR overview) |

**Limits (Web of Science):**

- **No restrictions: 1,203 results (02.09.2023)**

**SCOPUS:**

| **Concept** | **Search Terms** |
| --- | --- |
| **INDIGENOUS** | TITLE-ABS-KEY ( "indigenous" OR "aboriginal*" OR “native” OR Amerindian*) |
| **HEALTH** | TITLE-ABS-KEY ( health OR "one health" OR wellness OR wellbeing OR "well-being" OR disease OR morbidity OR mortality OR illness OR "infect*" OR death OR injur* OR medical OR disorder ) **AND** |
| **CLIMATE CHANGE** | TITLE-ABS-KEY ( "climate change" OR "climatic change" OR "environmental change" OR "environmental loss" OR "environmental degradation" OR "environmental dispossession" OR "changing climate" OR "ecosystem change" OR "ecological change" OR "climate risk" OR "climatic risk" OR "extreme climate" OR "climate* uncertaint*" OR "climate variability" OR "climatic variability" OR "climate disaster" OR “climate resilience” OR "carbon footprint" OR "global warming" OR “earth warming” OR “global temperature” OR "greenhouse effect" OR "greenhouse gas*" OR "GHGE" OR "carbon emission" OR carbon OR decarbonization OR holocene OR anthropocene OR "cryospher*" OR biodiversity OR {biodiversity loss} ) **AND** |
| **REVIEW OF REVIEWS** | TITLE-ABS-KEY ( review* OR {metaanalysis} OR {meta analysis} OR {knowledge synthesis} OR {evidence synthesis} OR {overview} ) |

**Limits (Scopus):**

- English ( LIMIT-TO ( LANGUAGE , "English" ) )
- Journals ( LIMIT-TO ( SRCTYPE , "j" ) )

**PubMed:**

| **Concept** | **Search Terms** |
| --- | --- |
| **INDIGENOUS** | (Indigenous people[MeSH] OR Indians, North American[MeSH] OR Health Services, Indigenous[MeSH] OR indigenous[tw] OR aboriginal*[tw] or Native[tw] OR Amerindian*[tw])  **AND** |
| **CLIMATE CHANGE** | (Climate change[MeSH] OR climate chang*[tw] OR global warming[tw] OR greenhouse effect[tw] OR greenhouse gas*[tw] OR biodiversity[tw] OR carbon footprint[tw] OR climate resilience[tw] OR earth warming[tw] OR global temperature[tw])  **AND** |
| **REVIEW OF REVIEWS** | (Review[ptyp] OR meta analysis[ptyp] OR review*[tiab] OR meta analysis[tiab] OR metaanalysis[tiab] OR knowledge synthesis[tiab] OR evidence synthesis[tiab] OR overview[tiab]) |

**Limits (PubMed):**

- **No restrictions: 876 results (02.09.23)**

**INDIGENOUS**

| **[tw] = title, abstract, MeSH** | **MeSH subject headings** |
| --- | --- |
| Indigenous  Aboriginal*  Native  Amerindian* | indigenous people  Indians, North American  Health Services, Indigenous    Expanded MeSH terms:  **Indigenous Peoples**  [American Indian or Alaska Native](https://www.ncbi.nlm.nih.gov/mesh/68044467)  [Indians, Central American](https://www.ncbi.nlm.nih.gov/mesh/68007197)  [Indians, North American](https://www.ncbi.nlm.nih.gov/mesh/68007198) +  [Indians, South American](https://www.ncbi.nlm.nih.gov/mesh/68007199)  [Australian Aboriginal and Torres Strait Islander Peoples](https://www.ncbi.nlm.nih.gov/mesh/2103394)  [Maori People](https://www.ncbi.nlm.nih.gov/mesh/2103395)  [Native Hawaiian or Other Pacific Islander](https://www.ncbi.nlm.nih.gov/mesh/68044468)    **+Indians, North American**  [Alaska Natives](https://www.ncbi.nlm.nih.gov/mesh/2016806)  [Indigenous Canadians](https://www.ncbi.nlm.nih.gov/mesh/2052192)  [Inuit](https://www.ncbi.nlm.nih.gov/mesh/68004930)  [Navajo People](https://www.ncbi.nlm.nih.gov/mesh/2103334)  [Pima People](https://www.ncbi.nlm.nih.gov/mesh/2103335)    No MeSH for African Indigenous Peoples; European Indigenous Peoples (e.g., Sami) |
| Indigenous people[MeSH] OR Indians, North American[MeSH] OR Health Services, Indigenous[MeSH] OR indigenous[tw] OR aboriginal*[tw] or Native[tw] OR Amerindian*[tw] | |

**CLIMATE CHANGE**

| **[tw] = title, abstract, MeSH** | **MeSH subject headings** |
| --- | --- |
| Climate chang*  Global warming  Greenhouse effect  Greenhouse gas*  Biodiversity  Carbon footprint  Climate resilience  Earth warming  Global temperature | Climate change    **Climate Change**  [Global Warming](https://www.ncbi.nlm.nih.gov/mesh/68057232)  [Sea Level Rise](https://www.ncbi.nlm.nih.gov/mesh/2030999) |
| Climate change[MeSH] OR climate chang*[tw] OR global warming[tw] OR greenhouse effect[tw] OR greenhouse gas*[tw] OR biodiversity[tw] OR carbon footprint[tw] OR climate resilience[tw] OR earth warming[tw] OR global temperature[tw] | |

**REVIEW OF REVIEWS**

| **[tiab] = title/abstract only** | **[ptyp] = publication type** |
| --- | --- |
| review*  meta analysis  metaanalysis  knowledge synthesis  evidence synthesis  overview | Review[ptyp]  meta analysis [ptyp] |
| Review[ptyp] OR meta analysis[ptyp] OR review*[tiab] OR meta analysis[tiab] OR metaanalysis[tiab] OR knowledge synthesis[tiab] OR evidence synthesis[tiab] OR overview[tiab] | |

**CINAHL (EBSCOHost):**

| **Concept** | **Search Terms** |
| --- | --- |
| **INDIGENOUS** | ("indigenous" OR "aboriginal*" OR “native” OR Amerindian*)  **AND** |
| **CLIMATE CHANGE** | ("climate change" OR "climatic change" OR "environmental change" OR "environmental loss" OR "environmental degradation" OR "environmental dispossession" OR "changing climate" OR "ecosystem change" OR "ecological change" OR "climate risk" OR "climatic risk" OR "extreme climate" OR "climate* uncertaint*" OR "climate variability" OR "climatic variability" OR "climate disaster" OR “climate resilience” OR "carbon footprint" OR "global warming" OR “earth warming” OR “global1 temperature” OR "greenhouse effect" OR "greenhouse gas*" OR "GHGE" OR "carbon emission" OR carbon OR decarbonization OR holocene OR anthropocene OR "cryospher*" OR biodiversity OR “biodiversity loss”) **AND** |
| **REVIEW OF REVIEWS** | (review* OR “metaanalysis” OR metaanalysis OR "knowledge synthesis" OR "evidence synthesis" OR overview) |

**Limits (CINAHL):**

- Academic Journals only (use left panel)
- **No date restrictions: 65 (02.09.23)**

**Campbell Collaboration** [hand searched]

| **Concept** | **Search Terms** |
| --- | --- |
| **INDIGENOUS** | (Indigenous OR aboriginal)  **AND** |
| **CLIMATE CHANGE** | (climate OR climate change OR environment)  **AND** |
| **HEALTH** | (health OR wellbeing OR wellness) |

- **No restrictions: 1 (02.09.23)**

**Additional File 2.2 –** Approach for hand searching of journals, to explore search hedge sensitivity for the database search

- Scan through every issue of every volume from the past 10 years (2013 – present), inclusive of 2013 (for *The Journal of Climate Change and Health; Environmental Health Perspectives;* and *The Lancet Planetary Health*) and without date restrictions (for the *International Journal of Circumpolar Health*; *Anthrosource; AlterNative;* and the *International Journal of Indigenous Health*)
- Look at **titles** only
- A title must include the one of the "**Indigenous** terms" AND one of the **health** terms AND one of the **climate change** terms AND one of the "**review of reviews**" terms from the search hedge used for databases.
- Any articles meeting this set of criteria will be cross-referenced with the records retrieved from the database search to see if it has been captured in the search

**Additional File 2.3 –** Examples of topics included/excluded

| Included | Excluded |
| --- | --- |
| - Articles focused on a geographic location, rather than a population, but where the **majority of the population is Indigenous** (e.g., Arctic; Pacific Islands; Amazon; “small island nations”; “mountainous areas”) - Articles on climate change impacts on **ecosystem services/ecosystem health**, where some connection is made to Indigenous Peoples' health - Articles on **community adaptation, participatory monitoring** of climate change impacts within the context of Indigenous health research | - Articles where **resource extraction** is the antecedent of health/wellbeing impacts (not climate change explicitly) - Review **“language”** in title/abstract but not using review methodology (e.g., reviewing concepts but not literature; literature scans)   - Historiographic reviews   - Computational linguistics - Record is a review **protocol**, not the review itself - Articles that report a **lit review among other methods** (e.g., lit review plus surveys, focus groups all reported in one article), whereby the other methods are prominent - Articles that review literature with a focus on **research methodology/conceptual approaches** in climate-health research (e.g., within its reviewed literature, the article assesses **research**, but does not review literature on the **topic** itself, and therefore does not directly contribute to addressing our research objectives) - Articles that focus on **overarching political processes or context** for a climate-health topic (e.g., focus on conservation governance). |

**Additional File 2.4 –** Data extraction tool for grey literature sources

**Adapted slightly from the published literature data extraction tool. Specific domains below may be iterated, depending on the type of grey literature record and associated relevance of the domains.*

| **Research Objective** | **Data Extraction Domains** |
| --- | --- |
| To characterize the extent, range, and nature of secondary literature on climate change, biodiversity loss, and Indigenous health and wellbeing globally | - Name of record - Year of publication - Organization or institution, if applicable - Geographic location(s) covered, if applicable - Indigenous Peoples (Nations, groups, organizations) - Type of record (e.g., working paper, report, policy brief, etc.) - Theory/framework/model engaged (e.g., EcoHealth, One Health, nature-based solutions) - Purpose/aim of the record |
| To examine the connections between climate change, biodiversity loss, and Indigenous health and wellbeing, characterizing the proximal, intermediate, and distal impacts within | - Relationships examined between concepts (e.g., climate change AND Indigenous health broadly; biodiversity loss AND Indigenous mental health) and rationale for this examination - Proximal impact(s) (if applicable): - Intermediate impact(s) (if applicable): - Distal impact(s) (if applicable): - Specific impacts on biodiversity - Scale of impact (e.g., individual, household, community, population, regional, national, global)   - Explanation - Key findings/conclusions about the relationships studied |
| To explore the gendered impacts of climate change and biodiversity loss on Indigenous health and wellbeing | - Summary of findings RE: gendered impacts - How sex/gender are discussed in this context |
| To identify responses to climate change and biodiversity loss that also advance Indigenous health and wellbeing | - Recommended strategies to address impacts; when applicable, categorize as:   - Community-level/population-level   - Regional-level/global-level   - Policy responses (if applicable)   - Future research |
